# Patterns of drug use among a sample of drug users and injecting drug users attending a General Practice in Iran

**DOI:** 10.1186/1477-7517-3-2

**Published:** 2006-01-24

**Authors:** Carolyn Day, Bijan Nassirimanesh, Anthony Shakeshaft, Kate Dolan

**Affiliations:** 1 National Centre in HIV Epidemiology and Clinical Research, University of New South Wales, Level 2, 376 Victoria Street, Darlinghurst, NSW 2010, Australia; 2 Persepolis Centre, 3^rd ^Floor, No: 902, Harandi Sq, Shoosh St, Tehran, Iran; 3 National Drug and Alcohol Research Centre, University of New South Wales, NSW 2052, Australia

## Abstract

**Aim:**

This study aimed to examine drug use, drug treatment history and risk behaviour among a sample of Iranian drug users seeking treatment through a general practice clinic in Iran.

**Methods:**

Review of medical records and an intake questionnaire at a large general practice in Marvdasht, Iran, with a special interest in drug dependence treatment. Records from a random sample of injecting drug users (IDU), non-injecting drug users (DU) and non-drug using patients were examined.

**Results:**

292 records were reviewed (34% IDU, 31% DU and 35% non-drug users). Eighty-three percent were males; all females were non-drug users. The mean age of the sample was 30 years. Of the IDU sample, 67% reported sharing a needle or syringe, 19% of these had done so in prison. Of those who had ever used drugs, being 'tired' of drug use was the most common reason for seeking help (34%). Mean age of first drug use was 20 years. The first drugs most commonly used were opium (72%), heroin (13%) and hashish/ other cannabinoids (13%). Three quarters reported having previously attempted to cease their drug use. IDU were more likely than DU to report having ever been imprisoned (41% vs 7%) and 41% to have used drugs in prison.

**Conclusion:**

This study has shown that there is a need for general practice clinics in Iran to treat drug users including those who inject and that a substantial proportion of those who inject have shared needles and syringes, placing them at risk of BBVI such as HIV and hepatitis C. The expansion of services for drug users in Iran such as needle and syringe programs and pharmacotherapies are likely to be effective in reducing the harms associated with opium use and heroin injection.

## Introduction

Opium has been consumed in Iran for at least three centuries [1]. Iran is on the main opium trade route from Afghanistan, although data on availability of opium and opium products in Iran is limited [1]. A rapid assessment of substance drug use in 1998/1999 found that three quarters of the illicit drug users interviewed used opium and more than third use heroin [2]. There is also some evidence of increased injecting drug use in Iran [2]. However, there is currently very little known about patterns of drug use and risks associated with injecting in Iran.

Injecting drug use is associated with a number of negative health outcomes, including increased mortality [3] and morbidity from overdose [4], increased risk of the drug dependence [5], and is of considerable public health significance due to the increased blood-borne viral infection (BBVI) transmission [6]. HIV infection has been found to be very high among IDUs, especially in those areas without widespread harm reduction initiatives [7]. Furthermore, experience in other countries has demonstrated that the incidence and prevalence of HIV infection can change rapidly among injecting drug users [8]. The prevalence of hepatitis C is high among populations of IDUs, and globally IDU represent the largest risk group for infection [9].

Research with Iranian volunteer blood donors found that those with a history of injecting drug use were 53 times more likely to be infected with HCV than other groups [10]. A recent study of drug dependent prisoners, revealed a HIV prevalence of 1.2% and a HCV prevalence of 47.4% [11]. In order to be optimally cost-effective, health care interventions aimed at reducing the spread of BBV should be tailored to the specific risk-factors of injecting drug users.

However, specific information on injecting drug users in Iran, including help seeking and blood-borne virus risk behaviour, is currently insufficient to allow such tailoring of health promotion and intervention strategies, and to facilitate the monitoring of risk-factor trends over time. Therefore the aim of this paper was to examine drug use, drug treatment history and risk behaviour among a sample of Iranian drug users seeking health care through a general practice clinic in Iran.

## Methods

The Marvdasht Clinic is a general practice clinic, with a special interest in drug dependence and treatment in Marvdasht, Shiraz, Iran. The clinic arranges general practice services and substance abuse treatment, especially pharmacotherapy for opioid dependence. The clinic also offers a drop in centre for drug users every day between 8.30 am and 7 pm.

Basic demographic information (age, gender) was collected and a physical examination performed on all those who attend the clinic. Those who reported any drug use also completed a brief drug use questionnaire administered by the clinician which was used in the assessment and treatment of the patient. Information was elicited from patients in Farsi and entered onto the forms in English. All data presented were collected by one clinician (BN).

A random selection of 200 substance abusing patient records (100 drug users and 100 injecting drug users) and 100 general patients' records were selected. Computer generated random numbers were used to select client records based on their registration number.

Clinic records were de-identified and data were entered into SPSS (version 11.0). Continuous data were analysed using *t *tests. Where data were highly skewed the Mann-Whitney U statistic was used and medians reported. Categorical data are presented as proportions and were analysed using the chi square test. All data were analysed using SPSS (version 11.0).

## Results

Three thousand patients attended the clinic between 1999 and 2002. Approximately 50% attended the clinic as general patients, 10% were injecting drug users and 40% were drug users but not injecting.

Two-hundred and ninety-two records were selected, of which 100 (34%) were completed on injecting drug users (IDU), 90 (31%) were completed on drug users who did not inject (DU) and 102 (35%) were completed on non-drug users (i.e. general clinic clients). The sample comprised 241 males (83%) and 30 females. All females were non-drug using patients. The mean age of patients was 30 years (SD 10.29). The drug using sample tended to be younger than the other clinic patients (27 years vs 35 years, *t*_289 _= 6.83, p < 0.001).

### Blood borne viruses

Blood-borne virus status was unknown for the majority of the patients. In the 32 cases where hepatitis B status was known, the six patients who were antibody positive, all were IDU. In the 20 cases where hepatitis C status was known, five patients (25%) were antibody positive and all were IDU. Only 19 patients had their HIV status recorded, of whom only one was positive, this patient was an IDU.

### HIV risk behaviour

The majority (67%) of IDU reported having shared a needle or syringe at some time. Sixty two patients reported the location where this took place and the most common was somewhere other than home (44%), in prison (19%) and at home (1%).

## Drug using clients only (n = 190)

### Drug use history

The mean age of first drug use was 20 years (SD 4.80). The IDUs tended to be younger at first drug use than the DUs (19 years vs 22 years, *t*_186 _= 5.08, p < .001).

Opium was the drug most commonly first used (72%), followed by heroin (13%) and hashish or another cannabinoid (13%). Four patients (2%) reported buprenorphine as the drug they first used.

Patients were most commonly with friends (38%) at the time of first drug use, with family (28%) or at work (22%). Fifteen patients (8%) reported first using drugs in military service and eight (4%) reported being in prison when they first used drugs.

Opium was the primary drug for half the patients (50%), followed by heroin (43%). A small proportion reported buprenorphine as their primary drug (6%).

### Reason for attending the clinic

The majority (73%) of drug using patients did not state a reason for attending the clinic. Of the remaining 51 patients who stated a reason for attending the clinic, the main reason given was related to family issues (36 patients), followed by 'compulsory testing' (11 patients) and court (3 patients).

### Family drug use history and support

A third of patients (31%) identified a family member who also used drugs. Of those 59 patients, a sibling (63%) or a parent (32%) were the most commonly identified relative. A history of drug use in the patient's parents' family was identified by 78 patients; this was most commonly identified in the patient's mother's family (65%).

The majority of drug using patients (92%) reported having drug using friends; 80% reported that they had at least two drug using friends. Of the 174 patients with drug using friends 131 (75%) reported still having contact with these drug using friends.

Only 10 patients (5%) reported having no family support. Of the 180 patients who reported having family support, 34% identified a sibling as providing this support, 32% identified their spouse, 31% identified a parent and four patients (2%) identified their child as providing support.

### Ceasing drug use

Patients were asked about the most important reason for ceasing their drug use. Being 'tired' (of using drugs) was the most common response (34%), followed by work reasons (21%; included having lost their job as a result of drug use), family (17%) and marriage (2%). 'Other' reasons were nominated by 40 patients, 10 of which were related to health, others included being sent for compulsory testing, having a driver's licence revoked or a work/study issue.

### Drug treatment

Almost three quarters of patients reported having previously attempted to cease their drug use. Of these, 11% had attempted to cease drug use once, while the majority (75%) had made several attempts and had done so a median of two times (range 1–10). There was no difference between the IDUs and DUs in terms of number of attempts to cease drug use.

Of the 146 patients who had attempted to cease drug use and received help, 36% received help from their spouse, 25% from a sibling, 19% from a friend and 18% from a parent. Twenty patients (12%) reported receiving no help or support during their attempts to cease drug use.

Patients reported using a variety of drugs to assist with drug use cessation in including homemade capsules (95%), drugs such as benzodiazepines, opiates such as black water opium, methadone, morphine and various other opium compounds (63%).

Of those who had previously attempted to cease drug use, 17 (10%) had remained abstinent for less than a week. The median number of weeks abstinent for the 147 patients who reported a week or more of abstinence was eight (range 1150;104). DU reported being abstinent for significantly more weeks than IDU (Mann-Whitney *U *1705, p < .001).

Reasons cited for patient relapse included health problems (55%), withdrawal (39%), drug craving (32%) and a range of other symptoms (26%) such as insomnia, loss of energy, anxiety and depression, pain, irritability and sexual dysfunction.

### Prison

Forty-five (24%) of the patients had a history of incarceration. IDU were more likely to have been to prison than DU (41% vs 7%, χ^2 ^= 29.7, p < 0.001). Forty-one patients (91% of those ever imprisoned) reported having used drugs while in prison. All DUs and 85% of IDU who reported having been to prison used drugs in prison. The drugs used in prison were opium (55%), heroin (20%) and hashish/ cannabis (9%). Only one patient reported receiving drug treatment in prison.

## Discussion

This study found that Iranian drug users seeking treatment for drug use problems most commonly use opium and heroin. Drug users presenting at this clinic tended to be younger than clients presenting with non drug-related problems. None of the drug using patients in this study were female. There is limited information on the prevalence of drug use among Iranian women, and it is unclear whether the lack of female drug users in this clinic sample is due to a very low prevalence of problematic drug use among women in Iran or that they are more likely to constitute a 'hidden' population and are unwilling to attend for treatment. Other studies of Iranian drug users also reported few females [e.g. [12]]. Reports from western countries has shown that drug use has been associated with greater stigmatisation, reduced service utilisation [e.g. [13]] and different treatment seeking behaviours [e.g. [14]] for women than men.

The majority of patients had previously attempted to cease drug use; traditionally drug treatment in Iran has comprised abrupt cessation without medication or slow decrease in heroin /opium dose [15]. This study has demonstrated that Iranian drug users are willing to seek medical treatment for their drug dependency. Buprenorphine has been shown to be effective in the treatment of Iranian heroin [12] and opium [15,16] users but needs to be more widely available, especially through general practice clinics, such as that described here as it may reduce stigmatisation for the user. It may be necessary to establish a special clinic for female drug users with female staff to encourage them to seek treatment.

Of the IDU sample, more than two thirds reported sharing needles and syringes and therefore placing themselves at considerable risk of HIV and hepatitis C infection. Needle and syringe programs have been shown to reduce the prevalence of HIV [17,18], and in settings with accessible needle and syringe program and health promotion initiatives, have also been shown to reduce needle and syringe sharing [19,20]. Although NSP programs have been introduced to Iran, the programs are small in number and still in their infancy.

A quarter of the drug users in the study had a history of imprisonment, with injectors much more likely to report this, almost all of whom reported injecting drug in prisons. Prisons have been identified as a high risk environment [21,22]. Almost half the sample of incarcerated IDUs in a recent Iranian study reported having ever shared a needle /syringe compared to 18% of non-incarcerated IDUs [23]. Moreover, only one patient reported receiving treatment for drug use in prison. The Iranian prison department is in the process of developing a strategic plan for prisons. Recent research has shown that the provision of methadone maintenance in prison can reduce injecting drug use and hepatitis C infection [24].

Drug treatment services provide a useful point for HIV and other BBVI monitoring [25,26]. Clinics should try to collect complete data for each patient attending the clinic in order to better inform the service. For example, for 73% of patients no reason was given for attending the clinic. It would be useful to know why clients come to clinics so their anticipated needs can be met and retention improved in treatment or with services. Moreover, routine collection of data from services, including small independent units such as that described in the current study, provides useful information for treatment outcome monitoring [27]. Similar Australian practices have, for example, been able to provide information on treatment outcomes in terms of decreased heroin use (as measured by self-report and urinalysis) and increased employment [28], and more recently report on the impact of methadone maintenance on hepatitis C incidence [29].

Finally, this study has shown that Iranian drug users seeking treatment through a GP clinic predominantly use opium and heroin. Of those who inject drugs, the majority has shared needles and syringes, placing them at risk of HIV, hepatitis B and hepatitis C. The expansion of services for drug users in Iran such as needle and syringe programs and pharmacotherapies such as buprenorphine are likely to be effective in reducing the harms associated with opium use and the increasing problem of heroin injection.

## Competing interests

Bijan Nassirimanesh was the director of the Marvdasht Clinic.

## Authors' contributions

All authors were responsible for study conception and design. C Day was responsible for the statistical analysis and led writing of the paper. B Nassirimanesh was responsible for the initial survey design, data collection and provided comments on the manuscript. A Shakeshaft and K Dolan supervised all aspects of the work and provided extensive comments on the manuscript.
